# Neurotrophin‐3 acts on the endothelial‐mesenchymal transition of heterotopic ossification in rats

**DOI:** 10.1111/jcmm.14150

**Published:** 2019-01-22

**Authors:** Jie Zhang, Liang Wang, He Cao, Nan Chen, Bin Yan, Xiang Ao, Huiyu Zhao, Jun Chu, Minjun Huang, Zhongmin Zhang

**Affiliations:** ^1^ Department of Orthopedics The Third Affiliated Hospital, Southern Medical University Guangzhou Guangdong PR China; ^2^ Academy of Orthopedics, Guangdong Province Guangzhou Guangdong PR China; ^3^ Department of Plastic Surgery The First Affiliated Hospital of Jinan University Guangzhou Guangdong PR China

**Keywords:** EndMT, heterotopic ossification, neuroendocrine, neurotrophin‐3

## Abstract

Despite the fact that extensive studies have focused on heterotopic ossification (HO), its molecular mechanism remains unclear. The endothelial‐mesenchymal transition (EndMT), which may be partially modulated by neuroendocrine cytokines is thought to play a major role in HO. Neurotrophin‐3 (NT‐3), which has neuroendocrine characteristics is believed to promote skeletal remodeling. Herein, we suggest that that NT‐3 may promote HO formation through regulation of EndMT. Here, we used an in vivo model of HO and an in vitro model of EndMT induction to elucidate the effect and underlying mechanism of NT‐3 on EndMT in HO. Our results showed that heterotopic bone and cartilage arose from EndMT and NT‐3 promoted HO formation in vivo. Our in vitro results showed that NT‐3 up‐regulated mesenchymal markers (FSP‐1, α‐SMA and N‐cadherin) and mesenchymal stem cell (MSC) markers (STRO‐1, CD44 and CD90) and down‐regulated endothelial markers (Tie‐1, VE‐cadherin and CD31). Moreover, NT‐3 enhanced a chondrogenesis marker (Sox9) and osteogenesis markers (OCN and Runx2) via activation of EndMT. However, both EndMT specific inhibitor and tropomyosin‐related kinase C (TrkC) specific inhibitor rescued NT‐3‐induced HO formation and EndMT induction in vivo and in vitro. In conclusion, our findings demonstrate that NT‐3 promotes HO formation via modulation of EndMT both in vivo and in vitro, which offers a new potential target for the prevention and therapy of HO.

## 1 INTRODUCTION

HO, characterized as ectopic bone formation in extraskeletal soft tissues is usually acquired after injuries and rarely derives from genetic mutations, such as fibrodysplasia ossificans progressive (FOP).^1^ Currently, anti‐inflammation, radiation and surgical excision are common therapies, which are usually accompanied by a high recurrence rate.^2^ Although histopathological studies have revealed that HO develops via endochondral ossification,^3^ the detailed cellular and molecular mechanism of HO remains unclear. Inappropriate progenitor cell activation is thought to contribute to ectopic bone formation.^4^

The identification of progenitor cells responsible for HO has been extensively explored and debated. However, the cellular origin of ectopic bone in HO remains controversial.^5^ Previous studies observed that tissue injuries induced local MSCs to differentiate into bone in HO, which suggests that MSC dysregulation serves as a common cellular mechanism of HO.^6^, ^7^, ^8^ Recently, EndMT has been proven to generate endothelial‐derived mesenchymal stem‐like cells that participate in heterotopic bone formation.^9^ EndMT occurs in response to inflammatory cytokines, such as bone morphogenetic protein (BMP)‐4 and transforming growth factor (TGF)‐β2 and results in the loss of adhesion between cells, changes in cell polarity and shape and generation of highly invasive and motile mesenchymal stem‐like cells. During EndMT, endothelial‐specific markers, such as tyrosine kinase with immunoglobulin‐like and EGF‐like 1 (Tie‐1), vascular endothelial cadherin (VE‐cadherin) and CD31 are reduced, but mesenchymal‐specific markers, such as fibroblast‐specific protein 1 (FSP‐1), neural cadherin (N‐cadherin) and α smooth muscle actin (α‐SMA), increase.^10^ Previous studies indicated that endothelial cells from FOP lesions undergo inflammation‐induced EndMT, acquire a mesenchymal stem‐like phenotype and subsequently differentiate into osteoblasts suggesting the critical role of EndMT in HO formation. Similarly, in a model of BMP‐4 and TGF‐β2‐induced HO, the conversion of endothelial cells to MSC‐like cells via EndMT have been observed.^9^, ^11^ Although the effect of EndMT on HO formation has been identified, unraveling the underlying regulation mechanism of EndMT in HO remains challenging.

Current studies have speculated that neuroendocrine cytokines may, in part, regulate the EndMT process.^12^, ^13^ NT‐3, a polypeptide growth factor involved in the proliferation, differentiation and apoptosis of neuronal and non‐neuronal cells is believed to have neuroendocrine characteristics.^14^ However, comprehensive evidence elucidating the role and mechanism of NT‐3 in EndMT is lacking. Although previous studies have demonstrated that NT‐3 is critical for nervous system development and maintenance, increasing evidence now suggests that NT‐3 also plays a major role in the skeletal system.^15^ For example, in a recent study, with its high affinity receptor (TrkC), NT‐3 was identified as an important osteogenic and angiogenic factor for promoting bony healing of the injured growth plate in rats.^16^, ^17^ Similarly, in a rat femoral distraction healing model, NT‐3 localization was observed in chondrocytes and osteoblasts during the endochondral ossification process.^18^ Besides, in mice rib fracture models, localization of NT‐3 was observed in osteoblast‐like cells and hypertrophic chondrocytes at the fracture callus.^19^ In addition, NT‐3 was shown to improve fracture healing in rats by regulating the tibia BMD, biomechanical indexes and bone formation.^20^ Moreover, as an autocrine and paracrine factor, NT‐3 was detected in osteoblast‐like cells and hypertrophic chondrocytes in fracture callus in a mouse fracture healing model.^18^ Although these previous studies have described the effect of NT‐3 on bone healing or remodeling, the potential function and mechanism of NT‐3 in the regulation of HO remain unknown. Based on previous work, we hypothesize that NT‐3 may act as a neuroendocrine cytokine involved in promoting HO formation through regulation of EndMT. We used an in vivo model of HO and an in vitro model of EndMT induction to elucidate the effect and underlying molecular mechanism of NT‐3 on EndMT in HO formation. We demonstrated that the formation of heterotopic cartilage and bone is caused by EndMT and suggested a potential mechanism that NT‐3 can promote the formation of HO through induction of EndMT both in vivo and in vitro*.*

## 2 MATERIALS AND METHODS

### 2.1 Animal treatment

All animal experimental protocols were approved by the Ethics Committee for Animal Research of Southern Medical University. Ninety‐six Sprague Dawley (SD) rats (6‐week‐old and regardless of gender) were purchased from the Laboratory Animal Center of Southern Medical University and subjected to an Achilles tenotomy using a posterior midpoint approach under aseptic conditions to induce HO.^21^ For gene expression and histological analysis of NT‐3, rats were killed at 4, 8 and 12 weeks after surgery (n = 6/time point); a control group was also killed (n = 6). For the EndMT study, 24 injured rats were randomly divided into four groups (n = 6/group): three groups received a sub‐cutaneous injection of recombinant NT‐3, BMP‐4 or TGF‐β2 (1.2 mg/mL, receiving 12 mg injection per week) (R&D Systems, Minneapolis, MN, USA) once a week surrounding the Achilles tendon. For the study of HO formation associated with EndMT, the injured rats were randomly divided into another four groups: the HO control, NT‐3, NT‐3+dorsomorphin and dorsomorphin groups (n = 6/group). For the study of NT‐3 on EndMT and HO formation, the remaining injured rats were randomly divided into another four groups: the HO control, NT‐3, NT‐3+GNF5837 and GNF5837 groups (n = 6/group). Rats received an injection of NT‐3 and GNF5837 (1 mg/kg) (Tocris Bioscience, Bristol, UK) weekly around the injured sites and dorsomorphin was intraperitoneally administered daily (1 mg/kg) (R&D Systems). All rats in the HO control group were administered a saline vehicle weekly. Twelve weeks after the Achilles tenotomy, all rats were killed and their limbs were collected for further study.

### 2.2 Micro‐CT analysis

Micro‐CT scans (µCT 80, Scanco Medical, Bruttisellen, Zurich, Switzerland) were performed on limbs harvested from the experimental and control groups. Specimens were scanned with a mean 20‐μm slice thickness under the conditions of 60 kV at 150 μA. HO formation was evaluated using reconstructed 3D images and ectopic bone volume was calculated.

### 2.3 Histological analysis

The Achilles tendons of rats collected from the experimental and control groups were fixed, decalcified, dehydrated and processed for paraffin embedding in 4‐μm‐thick sections. To visualize ectopic bone formation within the injured Achilles tendon, haematoxylin and eosin (H&E) (Sigma‐Aldrich, St. Louis, MI, USA) as well as Safranin O (Sigma‐Aldrich) and Fast Green (Sigma‐Aldrich) (SOFG) staining were performed. Quantitative analysis of ectopic calcified areas on the sections were measured by ImageJ software.

### 2.4 Immunohistochemistry and immunofluorescence staining

After deparaffinization and rehydration, sections were treated with 200 mg/mL proteinase K (Sigma‐Aldrich) for 15 minutes at 37°C to unmask the antigen. Sections for immunohistochemistry (IHC) analysis were treated with 3% hydrogen peroxide for 15 minutes and then blocked with 1% goat serum at room temperature for 1 hour. Then, sections were immunostained with primary antibodies against NT‐3 (1:100, Abcam, ab65804), TrkC (1:100, CST, 3376S) for IHC and Tie‐1 (1:100, Abcam, ab27851), FSP‐1 (1:100, Abcam, ab68124), osteocalcin (OCN) (1:100, Abcam, ab13420) and Sox9 (1:100, Abcam, ab185966) for immunofluorescence (IF). After incubation at 4°C overnight, a species‐matched Alexa Fluor 488‐, Alexa Fluor 594‐ or HRP‐labelled secondary antibody was used (1:500) at 37°C for 1 hour. For IHC, DAB (ZSGB‐Bio, Beijing, China) was used as the chromogen and haematoxylin was used to counterstain. For IF, sections were mounted with DAPI (Roche Applied Science, Indianapolis, IN, USA). All sections were observed under a microscope.

### 2.5 qRT‐PCR analysis

Total RNA was extracted from Achilles tendon tissues or rat aortic endothelial cells (RAOECs) at the indicated time points with TRIzol reagent (Life Technologies, Grand Island, NY, USA). cDNA was synthesized with the TaKaRa PrimeScript RT Reagent Kit (Takara Biotechnology Co. Ltd., Shija, Japan). qRT‐PCR was performed with the TaKaRa SYBR Premix Ex TaqⅡ kit according to the manufacturer's instructions (Takara). The primers purchased from Shanghai Biological Engineering are provided in Table S1. Data were calculated using the 2^−△△CT^ method and relative gene expression in the treatment groups is presented as fold change in relation to the control group.

### 2.6 Cell isolation and identification

RAOECs were isolated from SD rats as described previously.^22^ The intact aorta was excised, a 6‐0 suture was fixed on one end of the aorta and the inner surface of the aorta was flipped to the outside by drawing the suture through the lumen of the blood vessel with a blunt no. 5 suture needle. The turnover aorta was then cultured in endothelial cell medium (ECM) (ScienCell, Carlsbad, USA) at 37°C under 5% CO_2_. The medium was refreshed every two days. After one week, the number of adherent cells was observed under a microscope. The adherent cells were identified as endothelial cells by IF staining with CD31 and Tie‐1 (Figure S1A). Third passage endothelial cells were used for further experiments.

### 2.7 Induction of EndMT, chondrogenic differentiation and osteogenic differentiation

For the EndMT induction, RAOECs were cultured in ECM in the presence of BMP‐4 (100 ng/mL), TGF‐β2 (100 ng/mL), GNF4837(100 ng/mL) or NT‐3 (100 ng/mL) (R&D Systems) for 2 weeks. For chondrogenic differentiation, cells were cultured in chondrogenic medium supplemented with ITS Liquid Media Supplement (100×) (Sigma‐Aldrich) for two more weeks after EndMT induction. For osteogenic differentiation, RAOECs were further cultured in osteogenic medium supplemented with 50 μmol/L ascorbic acid, 0.1 μmol/L dexamethasone and 10 mmol/L β‐glycerol phosphate (Sigma‐Aldrich) after EndMT induction for another 2 weeks.

### 2.8 Cell viability assay

After the induction of EndMT for 48 hours, cell viability was quantified using a Cell Counting Kit‐8 (CCK‐8, Dojindo, Japan) assay according to the manufacturer's instructions. The number of viable cells in each well was measured at an absorbance wavelength of 450 nm.

### 2.9 Scratch and transwell migration assays

The migratory ability of ROAECs was measured using the scratch and transwell migration assays after EndMT for 48 hours. For the scratch assay, cells were plated in dishes and a scratch was made in the cell monolayer with a P200 pipette tip. The cells were then incubated under 5% CO_2_ at 37°C and photographed for up to 48 hours. Closure of the scratch area was analysed using Image‐Pro Plus 6.0 and the scratch healing rate was quantified by the percentage change in the scratch area. For the transwell migration assay, cells were seeded in the upper chamber and the lower chambers were filled with low‐serum ECM medium. After 48 hours, non‐migrated cells were removed and migrated cells on the lower side of the membrane were stained with crystal violet. Images were captured in four random fields.

### 2.10 Cell staining

ROAECs were grown in osteogenic or chondrogenic medium after EndMT induction. To detect matrix calcification and chondrocyte proteoglycans, Alizarin Red (Sigma‐Aldrich) and Alcian Blue (Sigma‐Aldrich) staining were performed on the cultures grown in chondrogenic or osteogenic medium for 14 days. The images were collected from three independent experiments.

### 2.11 Western blot analysis

RAOECs lysates from the experimental and control groups were collected and prepared for western blot. We incubated primary antibodies (1000‐fold) against Tie‐1 (Abcam, ab27851), VE‐cadherin (Abcam, ab205336), CD31 (Abcam, ab24590), N‐cadherin (Abcam, ab76011), FSP‐1 (Abcam, ab68124), α‐SMA (Abcam, ab5694), CD44 (Abacm, ab157107), CD90 (Abacm, ab225), OCN (Abcam, ab13420), Sox9 (Abcam, ab185966), Runx2 (CST, 12556S), STRO‐1(Abcam, ab108994) and GAPDH (ZSGB‐Bio, China) overnight at 4°C followed by incubation with an anti‐IgG horseradish peroxidase‐conjugated secondary antibody (1:4000) for 1 hour. Chemiluminescence was detected using an enhanced chemiluminescence system. GAPDH served as a loading control.

### 2.12 Statistical analysis

All in vitro data are representative of three independent experiments and each experiment was performed in triplicate. In vivo data represent n = 6 rats/group. SPSS 20.0 (SPSS, Chicago, IL, USA) was used for statistical analysis and all values are presented as the mean ± SD. The results were compared by one‐way ANOVA followed by the least significant difference post hoc test and Student's *t* test. *P* < 0.05 was considered statistically significant.

## 3 RESULTS

### 3.1 Heterotopic cartilage and bone in HO are induced by EndMT

**Figure 1:**
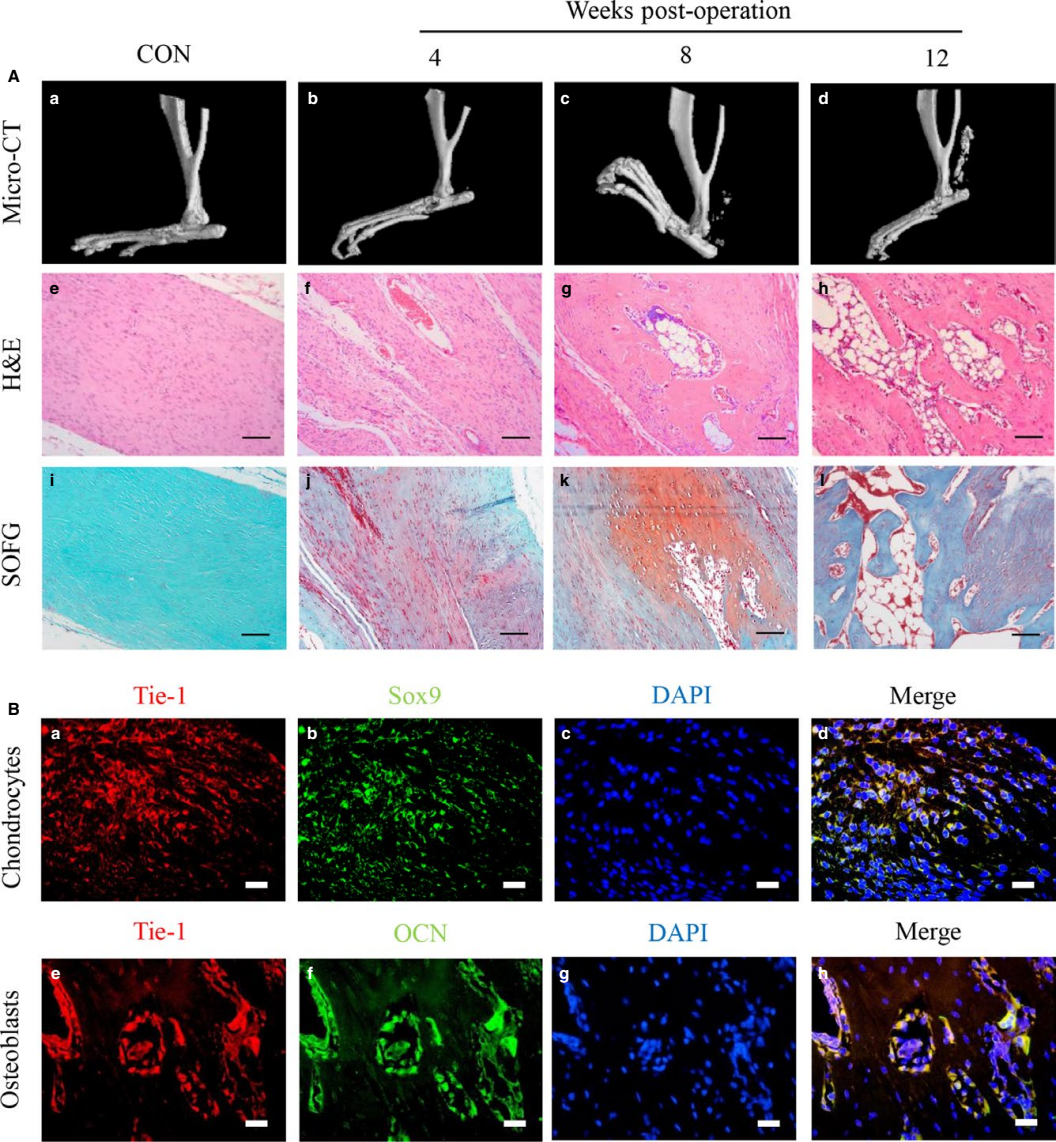
Heterotopic cartilage and bone in heterotopic ossification (HO) are induced by endothelial‐mesenchymal transition. (A) Micro‐CT images of HO formation in Achilles tendons in the normal control group (a) and at 4, 8 and 12 wk after surgery (b‐d). HO is visible beginning at 8 wk. Haematoxylin and eosin and SOFG staining of a normal Achilles tendon (e; i) and HO at 4, 8, and 12 wk after surgery (f‐h; j‐l). Proteoglycan (red) and ectopic bone (green). Scale bar, 100 µm. (B) Immunofluorescence analysis of HO lesions from Achilles tendons at 8 wk after surgery with antibodies specific for the endothelial marker Tie‐1 (red), the osteogenic marker osteocalcin (OCN) (green) and the chondrogenic marker Sox9 (green). Blue indicates DAPI staining of nuclei. Images show coexpression of the endothelial marker Tie‐1 and the chondrogenic marker Sox9 in chondrocytes (a–d) as well as coexpression of the endothelial marker Tie‐1 and the osteogenic marker OCN in osteoblasts (e–h). Scale bar, 20 µm. Representative images from one of the three experiments are shown (n = 6/group)

To examine the pathogenesis of HO, we utilized the rat Achilles tenotomy model to induce HO formation. Compared with that in the control group, heterotopic bone formation in the injured Achilles tendon began 4 weeks after the operation and continued growth was observed until 12 weeks as determined by micro‐CT observation (Figure 1A, 1‐d). Haematoxylin and eosin and SOFG staining illustrated a typical endochondral ossification process (Figure 1A, e‐h; i‐l). To determine whether heterotopic bone and cartilage at the injured Achilles tendon could be caused by EndMT, double IF staining was performed on paraffin sections collected from rat samples 8 weeks after tenotomy using antibodies specific for the endothelial marker Tie‐1, for the osteoblast marker OCN and for the chondrocyte marker Sox9. The results respectively showed the coexpression of endothelial and chondrogenic markers in chondrogenic lesions (Figure 1B, 1‐d) and the coexpression of endothelial and osteoblastic markers in osteogenic lesions (Figure 1B, e‐h). These results suggest that the formation of heterotopic bone and cartilage in HO arise from endothelial cells.

### 3.2 NT‐3 and TrkC are up‐regulated during HO formation

**Figure 2:**
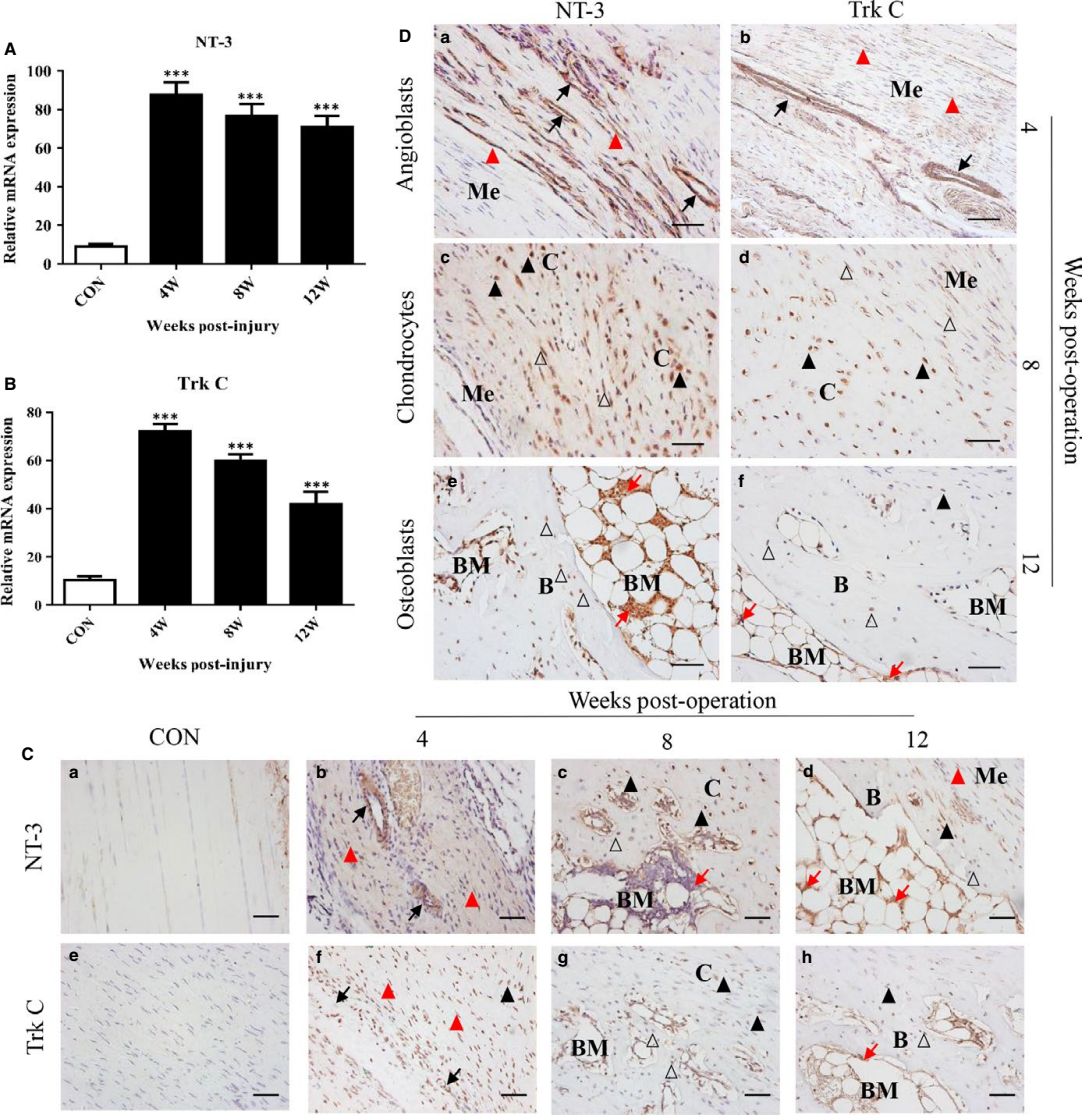
Neurotrophin‐3 (NT‐3) and tropomyosin‐related kinase C (TrkC) are up‐regulated during heterotopic ossification formation. (A, B) Gene expression of NT‐3 and TrkC in the control and experimental groups at 4, 8, and 12 wk after surgery. The mRNA levels (fold changes) of NT‐3 and TrkC were notably increased in the experimental groups versus those in the normal control group (n = 6/group). Scale bar, 100 µm. (C, D) Immunohistochemistry staining of NT‐3 and TrkC (brown) in the normal control group (C,a;e) compared with the groups (C,b–d;f–h & D,a–f) at 4, 8, and 12 wk after surgery. Immunohistochemistry showed that NT‐3 and TrkC were observed in vessel endothelial cells, infiltrating mesenchymal cells, chondrocytes, osteoblasts and newly formed bone marrow cells. B indicates bone, BM indicates bone marrow, C indicates cartilage, Me indicates mesenchyme, black arrows point to vessel endothelial cells, red triangles point to infiltrating mesenchymal cells, black triangles point to chondrocytes, blank triangles points to osteoblasts and red arrows point to newly formed bone marrow cells. The data represent the mean ± SD. ***P < 0.005 versus the normal control. Representative images from one of the three experiments are shown (n = 6/group)

To investigate the expression of NT‐3 and TrkC during HO formation, we profiled the mRNA levels of NT‐3 and TrkC in the injured Achilles tendons in rats; a normal Achilles tendon served as the control. Compared with those in the control group, the mRNA levels of NT‐3 were increased from 4 through 12 weeks after injury (Figure 2A). NT‐3 showed the highest induction at 4 weeks (exceeding 80‐fold), and its expression was gradually down‐regulated until 12 weeks (Figure 2A). Similarly, TrkC mRNA expression was significantly increased more than 70‐fold compared with that in the control group at 4 weeks (Figure 2B) and a progressive decline in its expression was observed during HO formation (Figure 2B).

To visualize the expression of NT‐3 and TrkC, we conducted IHC staining of NT‐3 and TrkC during HO formation. IHC staining of NT‐3 and TrkC was rarely found in the normal Achilles tendon (Figure 2C, a, e). For the injured Achilles tendon, NT‐3 and TrkC staining were positive from 4 to 12 weeks during ectopic bone formation (Figure 2C, b‐d, f‐h), which was consistent with the mRNA expression results. Moreover, NT‐3 and TrkC were obviously observed in vascular endothelial cells and infiltrating mesenchymal cells at 4 weeks (Figure 2D, a, b) and in chondrocytes and osteoblasts as well as in some newly formed bone marrow cells from 8 to 12 weeks (Figure 2D, 2‐f). These results demonstrate that NT‐3 and TrkC are significantly expressed during the HO formation phase in the injured Achilles tendon.

### 3.3 Effect of NT‐3 on the EndMT of HO

**Figure 3:**
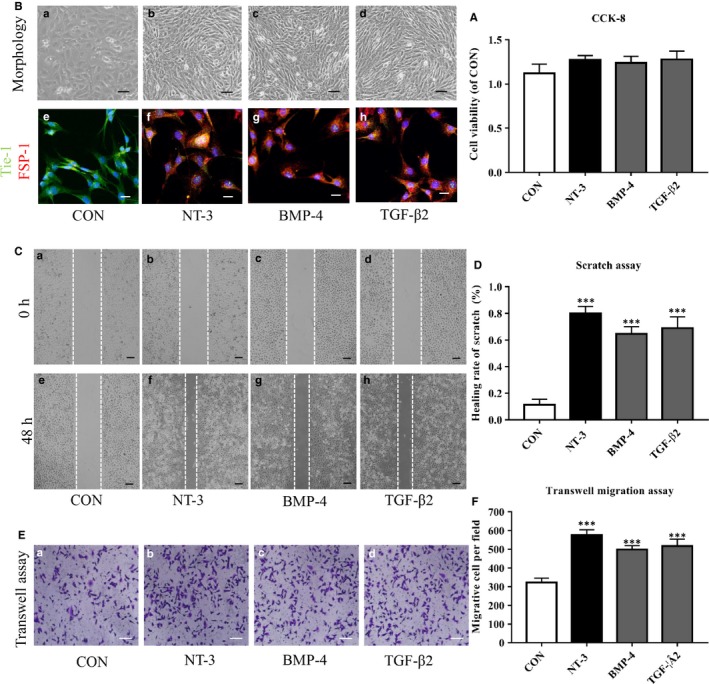
Neurotrophin‐3 (NT‐3) causes the transformation of cell morphology and acquisition of migratory function in rat aortic endothelial cells (RAOECs). Primary RAOECs were cultured in the presence or absence of NT‐3, BMP‐4 or TGF‐β2 for 48 hours. (A) Cell viability was assessed using the CCK‐8 assay. (B) Cell photography (top) showed a change from a cobblestone‐like endothelial cell morphology (a) to a fibroblast‐like morphology (b–d). Scale bar, 50 µm. Immunofluorescence analysis (bottom) showed the co‐staining of endothelial and mesenchymal markers (f–h; control group: e). Scale bar, 20 µm. Migratory function was evaluated using the scratch assay (C) and transwell migration assay (E) with quantitative analysis (D, F). Images of the scratch and transwell migration assays respectively, showing that NT‐3 increased RAOEC migration (C,b–d;f–h & E,b–d) versus that in the control group (C,a,e & E,a). Scale bar, 50 µm. Data in the figures represent the mean ± SD. ***P < 0.005 versus the control group. Representative images from one of three experiments are shown

BMP4 and TGF‐β2 are known to induce activin‐like kinase 2 (ALK2)‐dependent EndMT, which is involved in the formation of heterotopic bone and cartilage.^23^ To investigate the role of NT‐3 in EndMT in vitro, we induced EndMT in primary RAOECs with NT‐3, BMP‐4 or TGF‐β2. First, we carried out a CCK‐8 assay to investigate the potential toxicity and effects on cell proliferation. No significant difference in cell viability was observed between the different groups after 48 hours of treatment (Figure 3A), which indicated that stimulations with NT‐3, BMP‐4 or TGF‐β2 at 100 ng/mL had no toxicity on the growth of RAOCEs. Then, microscopic images of the experimental groups showed a marked change from a cobblestone‐like endothelial cell morphology to a fibroblast‐like morphology (Figure 3B, 3‐d). Co‐IF staining showed that the control group was positive for Tie‐1 but negative for FSP‐1 (Figure 3B, e), whereas NT‐3‐, BMP‐4‐ or TGF‐β2‐stimulated cells were positively stained for both proteins (Figure 3B, f‐h). We next performed scratch and transwell migration assays to elucidate the effect of NT‐3 on cell motility. The NT‐3 group notably promoted scratch closure compared with that in the control group after 48 hours of treatment and cells from the BMP‐4 and TGF‐β2 groups showed migration abilities similar to those in the NT‐3 group (Figure 3C,D). This effect of NT‐3 on RAOEC migratory function was further confirmed by a transwell migration assay and the results were consistent with those of the scratch assay (Figure 3E,F).

**Figure 4:**
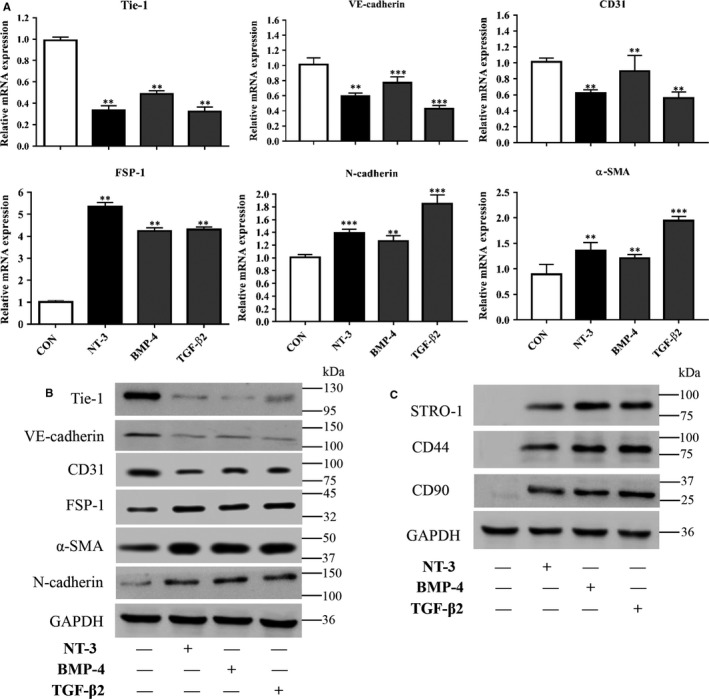
Neurotrophin‐3 (NT‐3) induces endothelial‐mesenchymal transition (EndMT) and an endothelial‐derived mesenchymal stem‐like phenotype. Primary rat aortic endothelial cells (RAOECs) were induced to undergo EndMT in the presence or absence of NT‐3, BMP‐4 or TGF‐β2 for 2 wk. Total RNA was then prepared for qPCR analysis and protein was extracted for western blot. qRT‐PCR showed that NT‐3 down‐regulated the expression of the endothelial markers Tie‐1, VE‐cadherin and CD31 and up‐regulated the expression of the mesenchymal markers FSP‐1, α‐SMA and N‐cadherin, which was consistent with the results of western blot (A, B). Immunoblotting also showed that the mesenchymal stem cells (MSC) markers STRO‐1, CD44 and CD90 were significantly expressed in the experimental groups (C). Each sample is representative of three experiments with similar results performed in triplicate. The data represent the mean ± SD. **P < 0.01 and ***P < 0.005 versus the control group

Subsequently, qRT‐PCR and western blot were conducted to analyse the expression of endothelial and mesenchymal markers in RAOECs after the induction of EndMT for 2 weeks. Down‐regulation of the endothelial markers Tie‐1, VE‐cadherin and CD31 and up‐regulation of the mesenchymal markers FSP‐1, α‐SMA and N‐cadherin were observed at both the gene and protein levels (Figure 4A,B). To determine whether RAOECs acquire a stem cell–like phenotype after EndMT induction, we next performed western blot with antibodies specific for MSC markers. Western blot showed no STRO‐1, CD44 and CD90 expression in untreated cells, whereas obvious expression of these molecules was observed in cells of the experimental groups (Figure 4C). Taken together, these results elucidate that NT‐3 can induce EndMT and acquisition of the mesenchymal stem‐like phenotype in EndMT‐induced RAOECs.

### 3.4 Effect of NT‐3 on bone formation in HO

**Figure 5:**
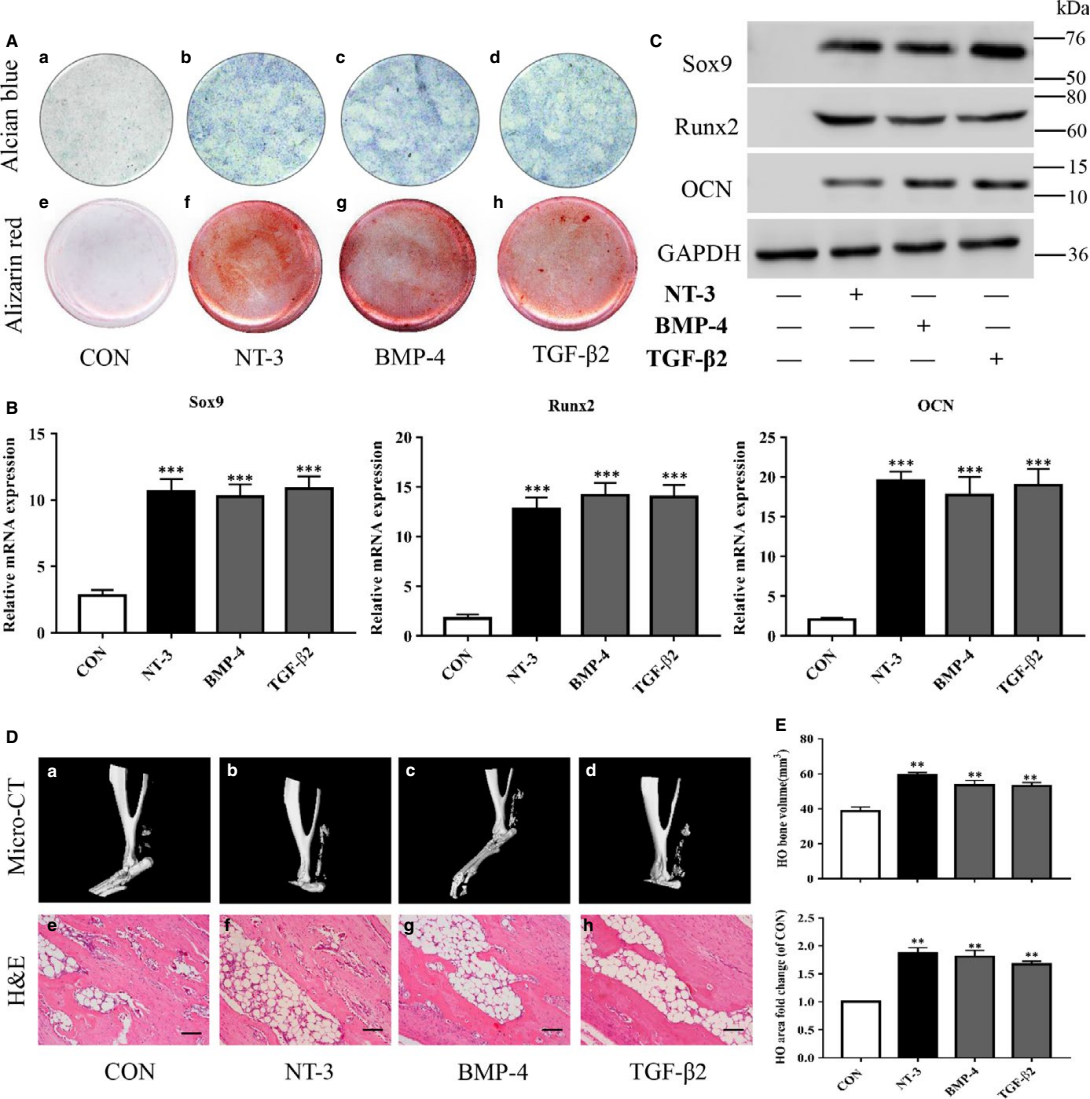
Effect of Neurotrophin‐3 (NT‐3) on bone formation in heterotopic ossification (HO). Primary rat aortic endothelial cells (RAOECs) were induced for endothelial‐mesenchymal transition with or without NT‐3, BMP‐4 or TGF‐β2 for 2 wk and further induced for chondrogenic and osteogenic differentiation for another 2 wk. (A) Cells in the experimental (b–d;f–h) and control group (a;e) were stained for Alcian Blue and Alizarin Red. (B) qRT‐PCR and (C) western blot both showed that NT‐3 enhanced the expression of the chondrogenic marker Sox9 and the osteogenic markers Runx2 and OCN. Each sample is representative of three experiments with similar results performed in triplicate. Recombinant NT‐3, BMP‐4 or TGF‐β2 was injected into the injured Achilles tendons of rats for 12 wk (n = 6/group). (D) Micro‐CT images of Achilles tendons and haematoxylin and eosin staining of HO with (E) quantitative analysis of bone volume and calcified areas showed increased HO formation in the experimental groups (D,b–d;f–h) versus that in the control group (D,a;e). The data represent the mean ± SD. **P < 0.01 and ***P < 0.005 versus the control group. Representative images from one of the three experiments are shown

Because MSCs are multi‐potent, we next investigated the function of NT‐3‐induced RAOECs on heterotopic bone formation in vitro. Cells were cultured to induce EndMT for two weeks followed by culture in chondrogenic or osteogenic differentiation medium for another 2 weeks. NT‐3, BMP‐4 and TGF‐β2 markedly enhanced Alcian Blue and Alizarin Red staining (Figure 5A) and significantly up‐regulated the expression of the chondrocyte marker Sox9 and the osteoblast markers Runx2 and OCN at both the gene and protein levels (Figure 5B,C). By contrast, neither cell staining nor these markers were detected in the control group.

To further determine the role of NT‐3 in HO formation in vivo, micro‐CT and haematoxylin and eosin staining were conducted to evaluate heterotopic bone formation. The ectopic bone volume and calcified area in the NT‐3‐injected Achilles tendon were obviously larger than those in the control group (Figure 5D, a,b; e,f), which was similar to the results found in the BMP‐4 and TGF‐β2 groups (Figure 5D, 5,D; g,h). In addition, quantitative analysis of the bone volume and calcified HO area suggested that NT‐3 significantly increased both bone volume and the calcified area of the injured Achilles tendon vs those in the control (Figure 5E). Taken together, these results elucidate that NT‐3 may contribute to heterotopic bone formation associated with EndMT confirming the involvement of EndMT and the role of NT‐3 in HO.

### 3.5 Rescue effect of Dorsomorphin on the induction of EndMT and HO formation by NT‐3

**Figure 6:**
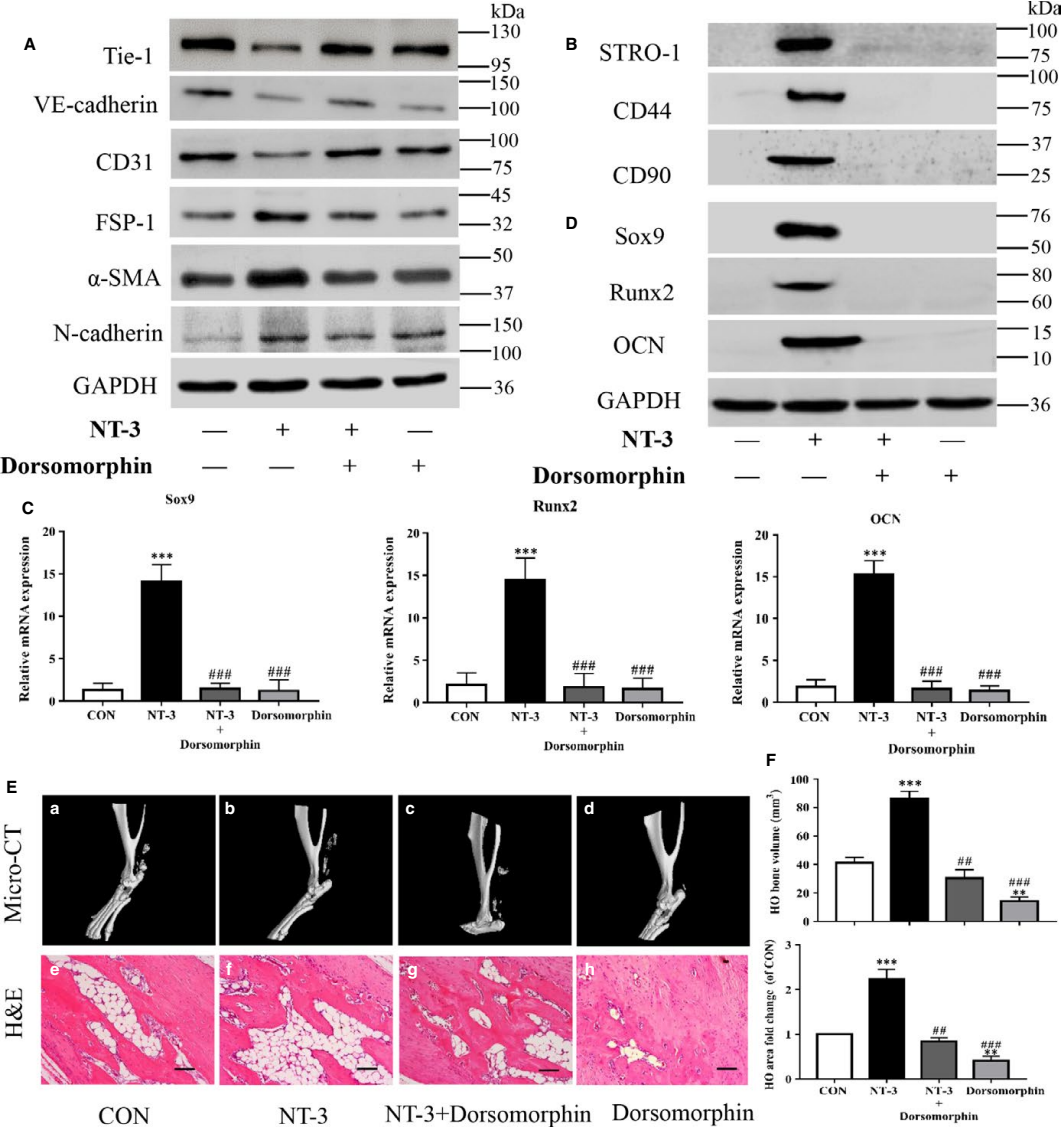
Dorsomorphin rescues the promotional effect of Neurotrophin‐3 (NT‐3) on endothelial‐mesenchymal transition (EndMT) induction and heterotopic ossification (HO) formation. Rat aortic endothelial cells (RAOECs) were induced for EndMT with NT‐3 in the presence or absence of dorsomorphin for 2 wk and further induced for chondrogenic and osteogenic differentiation for another 2 wk. (A, B) Western blot showed that dorsomorphin reversed the NT‐3‐induced expression of endothelial markers, mesenchymal markers and mesenchymal stem cells (MSC) markers. (C, D) qRT‐PCR and immunoblotting also showed a reduction in chondrogenic and osteogenic markers in the dorsomorphin‐treated groups. Each sample is representative of three experiments with similar results performed in triplicate. NT‐3 in the presence or absence of dorsomorphin was injected into the injured Achilles tendons of rats for 12 wk (n = 6/group). (E) Micro‐CT images of Achilles tendons and haematoxylin and eosin staining of HO with (F) quantitative analysis of bone volume and calcified areas showed that dorsomorphin (E,c–d;g–h) rescued the promotional effect of NT‐3 on HO formation (E,a–b;e–f). The data represent the mean ± SD. **P < 0.01 and ***P < 0.005 versus the control group. ^##^ *P* ＜ 0.01 and ^###^ *P* < 0.005 versus the NT‐3 group. Representative images from one of three experiments are shown

Dorsomorphin is known to inhibit the EndMT process,^24^ therefore western blot was performed to evaluate the effect of dorsomorphin on NT‐3‐induced EndMT in RAOECs. Dorsomorphin attenuated the NT‐3‐induced down‐regulation of endothelial markers and up‐regulation of mesenchymal markers (Figure 6A). Consequently, dorsomorphin also suppressed the NT‐3‐induced expression of the MSC markers STRO‐1, CD44 and CD90 (Figure 6B). To determine the role of dorsomorphin in NT‐3‐accelerated heterotopic bone formation in vitro, the expression of chondrogenic and osteogenic markers was assessed using western blot and qRT‐PCR. Dorsomorphin notably reduced the NT‐3‐induced expression of Sox9, Runx2 and OCN at both the gene and protein levels (Figure 6C,D). To further investigate the effect of dorsomorphin on NT‐3‐induced HO formation in vivo, micro‐CT and haematoxylin and eosin staining were performed to assess the ectopic bone formation in rats. Dorsomorphin rescued the NT‐3‐promoted formation of HO and the ectopic bone and calcified area obviously shrank compared to that in the NT‐3 treated group (Figure 6E). Quantitative analysis of the bone volume and calcified area confirmed the similar findings showing that dorsomorphin significantly reversed the increment of bone volume and calcified area induced by NT‐3 (Figure 6F). These data suggested that the effect of NT‐3 on the induction of EndMT and bone formation in HO can be attenuated by the EndMT‐specific inhibitor dorsomorphin.

### 3.6 Inhibition of NT‐3 suppressed the induction of EndMT and bone formation in HO

**Figure 7:**
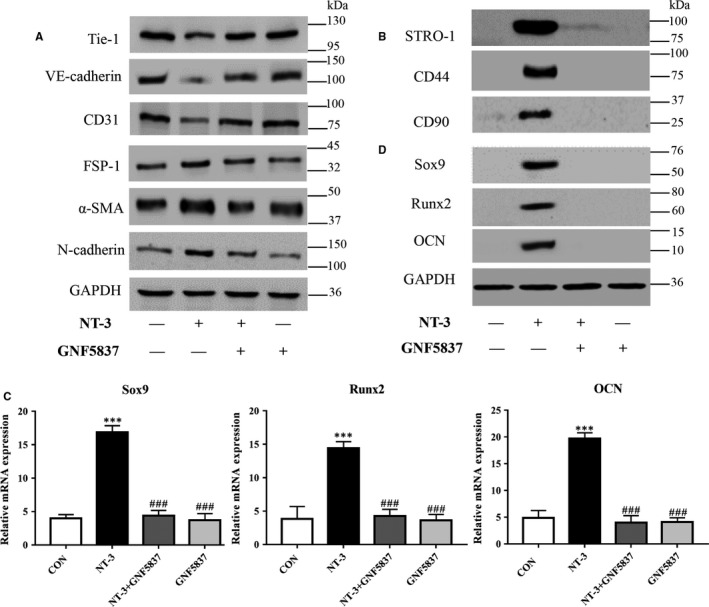
Inhibition of Neurotrophin‐3‐tropomyosin‐related kinase C (NT‐3‐TrkC) pathway suppressed endothelial‐mesenchymal transition (EndMT) induction in heterotopic ossification. Rat aortic endothelial cells (RAOECs) were induced for EndMT with Neurotrophin‐3 (NT‐3) in the presence or absence of GNF5837 for 2 wk and further induced for chondrogenic and osteogenic differentiation for another 2 wk. (A, B) Western blot showed that GNF5837 reversed the NT‐3‐induced expression of endothelial markers, mesenchymal markers and mesenchymal stem cells (MSC) markers. (C, D) qRT‐PCR and western blot also showed a reduction in chondrogenic and osteogenic markers in the dorsomorphin‐treated groups. Each sample is representative of three experiments with similar results performed in triplicate. The data represent the mean ± SD. ***P < 0.005 versus the control group. ^###^ *P* < 0.005 versus the NT‐3 group. Representative images from one of three experiments are shown

As NT‐3 and its TrkC receptor were observed during formation of HO (Figure 2), thus we tried to further explore role of NT‐3 on the induction of EndMT in HO. GNF5837, the inhibitor of TrkC receptor^25^ rescued down‐regulation of endothelial markers and up‐regulation of mesenchymal markers induced by NT‐3 (Figure 7A). In addition, GNF5837 suppressed the NT‐3‐induced expression of the MSC markers STRO‐1, CD44 and CD90 (Figure 7B) as well. Furthermore, GNF5837 markedly reduced the protein and gene expression of NT‐3‐induced Sox9, Runx2 and OCN (Figure 7C, 7), which was similar with the effect of Dorsomorphin.

**Figure 8:**
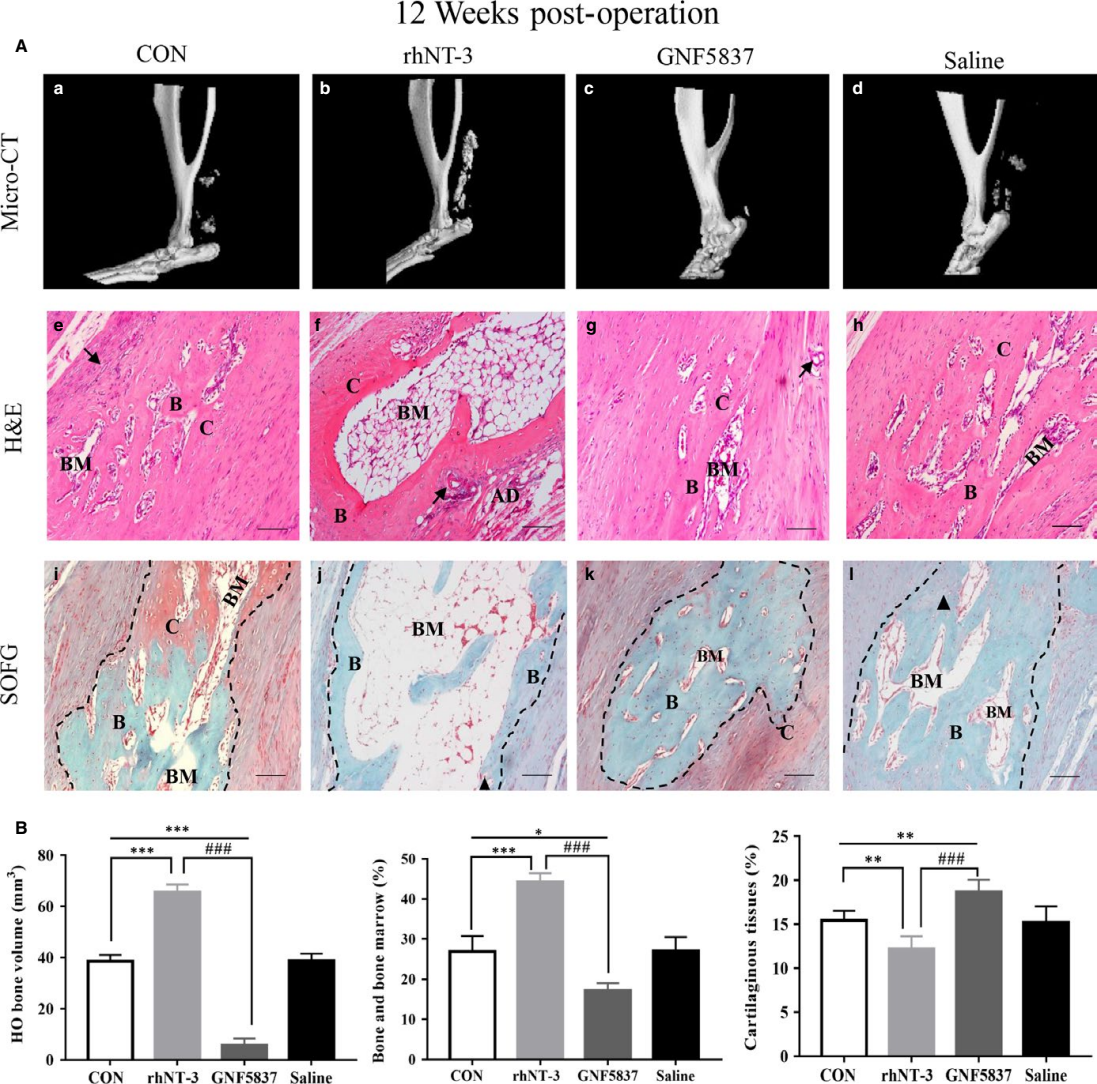
Inhibition of Neurotrophin‐3‐tropomyosin‐related kinase C (NT‐3‐TrkC) pathway suppressed heterotopic ossification (HO) formation. (A) Micro‐CT 3D reconstructed images of heterotopic bone at injured Achilles tendon of normal HO control rats (a), rhNT‐3‐treated rats (b), GNF5837‐treated rats (c) and saline‐triangle rats (d) at 12 wk. haematoxylin and eosin (e‐h) and SOFG (i‐l) staining of injured Achilles tendons and heterotopic ossification from different groups mentioned above. B indicates bone, BM indicates bone marrow, C indicates cartilage, black arrows point to stained blood vessels and black triangles point to stained chondrocytes. Dashed line of SOFG staining sections showed the newly formed heterotopic bone. Scale bar, 100 µm; (B) HO bone volume (mm^3^) at injured Achilles tendons (left); Quantitative histological analyses of proportions (%) of bone and bone marrow (middle) as well as cartilaginous tissues (right) of injured Achilles tendon at 12 wk. Representative images from one of three experiments are shown (n = 6/group). All data represent the mean ± SD. *P < 0.05, **P < 0.005 and ***P < 0.001 versus the HO control group. ^###^ *P* < 0.001 versus the rhNT‐3 group

To further illustrate the effect of NT‐3 on bone formation in HO, both rhNT‐3 and GNF5837 treatments were utilized with the injured Achilles tendons using the HO group and saline treatment as controls. In comparison with the HO control group and saline group at 12 weeks by micro‐CT scanning, rhNT‐3 obviously promoted HO formation whereas GNF5837 showed the opposite effects (Figure 8A, 8‐d; B) Additionally, haematoxylin and eosin and SOFG staining were conducted to calculate the proportions of different types of tissues (Figure 8A, e‐l; 8). The results showed that rhNT‐3 treatment significantly increased the amount of cancellous bone and bone marrow‐like tissues but reduced some cartilaginous tissues at 12 weeks. In the GNF5837 group, cancellous bone and bone marrow‐like tissues decreased when compared to either rhNT‐3 group or the HO control and saline treatment. Additionally, cartilaginous tissues in the rhNT‐3 group were reduced when compared to the HO control and saline treatment, whereas the percentage of cartilaginous tissues in GNF5837 treatment increased. Moreover, no significant difference was observed between the HO control and saline treatment either in micro‐CT or in histological staining indicating that administration of saline around the injured sites may not promote HO. Taken together, these data suggested that inhibition of NT‐3 could suppress EndMT induction and HO formation at injured Achilles tendons.

## 4 DISCUSSION

Although the cellular progenitors that lead to HO are yet to be identified, MSCs from local tissues, such as the Achilles tendon or skeletal muscle interstitium are logical candidates.^6^, ^8^, ^26^, ^27^ Based on Cre/loxP lineage tracing technology, endothelial progenitor cells were identified as one of the major contributors to HO formation. Recent studies have revealed that EndMT was induced in response to constitutive BMP signalling in animal models representing a dedifferentiation of endothelial cells to a mesenchymal stem‐like phenotype, which can subsequently redifferentiate into various cell lineages.^9^, ^23^, ^28^ Previous studies have also found that ectopic bone and cartilage cells express endothelial markers in a model of BMP‐4 and TGF‐β2‐induced HO, which suggests that heterotopic bone and cartilage formation arises from endothelial cells.^9^, ^11^ Additionally, these studies have confirmed the role of EndMT in either traumatic HO or in models of FOP and have demonstrated that endothelial progenitor cells underwent EndMT to generate MSC‐like cells that could be induced for osteogenic differentiation and chondrogenic differentiation. Similarly, our data demonstrated that chondrocytes and osteoblasts from HO lesions stain positive for endothelial‐specific markers implying the endothelial origin of HO in our animal models. Moreover, the fact that osteoblasts and chondrocytes from normal bone and cartilage do not express endothelial markers suggests that osteoblasts and chondrocytes in heterotopic bone or cartilage are produced by EndMT.^9^ Additionally, our data showed the transition from endothelial cells into mesenchymal stem‐like cells from many aspects including morphology, migratory function and the expression of genes and proteins of EndMT and MSC markers. Furthermore, endothelial cells induced to undergo EndMT significantly expressed the chondrogenic and osteogenic markers after the further osteogenic and chondrogenic differentiation. Taken together, these results confirm that EndMT generates mesenchymal stem‐like cells that can subsequently differentiate into chondrocytes and osteoblasts during the early stage of HO formation, which suggests the crucial role of EndMT in HO and provides new insights for prevention and therapy in HO management.

Despite the fact that extensive studies have illustrated the function of EndMT in HO, no studies have identified the potential regulation mechanism of EndMT. In recent studies, neuroendocrine cytokines were speculated to partially mediate EndMT progression.^12^, ^13^ Neuroendocrine cytokines have been demonstrated to be involved in the maintenance of the skeletal system, as they directly regulate osteoblastic function.^29^, ^30^ In the past, several studies have reported that NT‐3, a key molecule for the development and maintenance of the nervous systems^31^ acts as a neuroendocrine regulator in the neoangiogenesis of ischemic muscles, remodeling and repair of injured skin as well as wound healing in diabetes mellitus patients.^14^, ^29^, ^32^ However, detailed reviews that elucidate the role and regulation mechanism of NT‐3 in EndMT are still absent. Interestingly, increasing evidence implies the important neuroendocrine role of NT‐3 in regulating skeletal system remodeling.^12^, ^33^, ^34^ Previously, NT‐3 treatment was found to accelerate osteogenic differentiation in periodontal ligament cells and cementoblasts.^35^ In addition, a previous study demonstrated that a local application of NT‐3 accelerated fracture healing in rats.^20^ Furthermore, NT‐3 together with its receptor TrkC have been regarded as an osteogenic and angiogenic factor to promote the osteogenesis, vascularization and healing of bone and cartilage repair.^17^, ^32^, ^36^ However, none of these studies have revealed an erroneous regulation mechanism of NT‐3 associated with heterotopic bone formation. In the present study, our data showed NT‐3 and TrkC expression in vascular endothelial cells, chondrocytes, osteoblasts and some newly formed bone marrow cells at the Achilles tendon injured sites, which suggests that endogenous NT‐3 participates in ectopic bone formation. In addition, administration of exogenous NT‐3 induced an elevation in heterotopic bone volume at the Achilles tendon suggesting that NT‐3 can promote HO formation. Moreover, our data showed that NT‐3 up‐regulated mesenchymal markers and down‐regulated endothelial markers in primary RAOECs suggesting that NT‐3 can induce EndMT. Furthermore, our data showed that NT‐3 accelerated the chondrogenic and osteogenic differentiation of mesenchymal stem‐like cells acquired from the EndMT process indicating that NT‐3 can promote HO formation via mediation of EndMT. Taken together, our investigation identified for the first time the positive effect of NT‐3 on the induction of EndMT and the formation of HO. Although the relevant modulation mechanisms of NT‐3 in the promotion of HO formation may be involved in its autocrine or paracrine loop function,^19^, ^32^ the precise role of NT‐3 in HO formation was still absent. Moreover, the underlying regulation mechanism of NT‐3 in the induction of EndMT remains unelucidated as well. Thus, further studies are needed to investigate the potential mechanism of NT‐3 in HO formation associated with EndMT.

To further elucidate the precise function of NT‐3 on HO formation associated with EndMT, the specific inhibitors of were selected for studies. Dorsomorphin, which is identified as a specific inhibitor of EndMT^24^ attenuated the effect of NT‐3 on EndMT induction and HO formation promotion. Stimulation with dorsomorphin in vitro reversed the NT‐3‐induced EndMT by up‐regulating endothelial markers and down‐regulating mesenchymal and MSC markers. For HO formation in vitro, dorsomorphin reduced NT‐3‐induced chondrogenic and osteogenic differentiation at both the gene and protein levels. Similarly, in vivo, dorsomorphin rescued the NT‐3‐induced acceleration of ectopic bone formation. In addition, the specific inhibitor of TrkC (GNF5837) exhibited the similar rescue effects as dorsomorphin in NT‐3‐induced EndMT as well as HO formation. Interestingly, our in vivo data showed that dorsomorphin also reversed the bone volume and calcified areas without NT‐3 treatment indicating that dorsomorphin may at least partly prove beneficial in preventing EndMT‐dependent HO. Taken together, our findings revealed the rescue effects of dorsomorphin on EndMT as well as HO formation at the injured Achilles tendons, which may provide a new therapeutic preventive direction for the clinical management of HO.

Although we have observed that endogenous NT‐3 and TrkC were notably induced at the sites of HO, unfortunately, the potential sources of endogenous NT‐3 in this study remained unknown. We rarely found the expression of endogenous NT‐3 (Figure 2a) and TrkC (Figure 2e) in the normal Achilles tendons suggesting that NT‐3 may be not produced by the resident cells of normal Achilles tendons. It is reported that, apart from nervous system and skeletal diseases, NT‐3 has been regarded as a clinical indicator and can be elevated in various disorders occurred in skin, lung and ovary. NT‐3 can be examined from serum either in patients with inflammation or normal people^37^ indicating the presence of endogenous NT‐3 and its activation by inflammation. Additionally, the presence of NT‐3 and TrkC at the injured sites suggested that NT‐3‐TrkC signalling pathway may participate in HO formation as well. Our data suggested that blocking of NT‐3 by inhibiting its TrkC receptor could alleviate HO formation and suppress EndMT induction. However, further study is required to focus on the possible origin of endogenous NT‐3 and NT‐3‐TrkC signalling pathway to explore more precise mechanism of NT‐3 in HO formation associated with EndMT. Moreover, previous studies have reported that dorsomorphin could block EndMT induced by BMP‐4 in patients with FOP due to the ability of inhibiting ALK‐2/Smad signalling pathway^9^, ^10^ suggesting that the BMP/ALK‐2/Smad signalling pathway may play a crucial role in HO formation. Apart from inhibition of EndMT, treatment of dorsomorphin may reduce the development of HO through inhibition of BMP/ALK‐2/Smad signalling pathway in this study. Therefore, further researches should be performed to illustrate the possible relationships between EndMT and BMP/ALK‐2/Smad signalling pathway.

**Figure 9:**
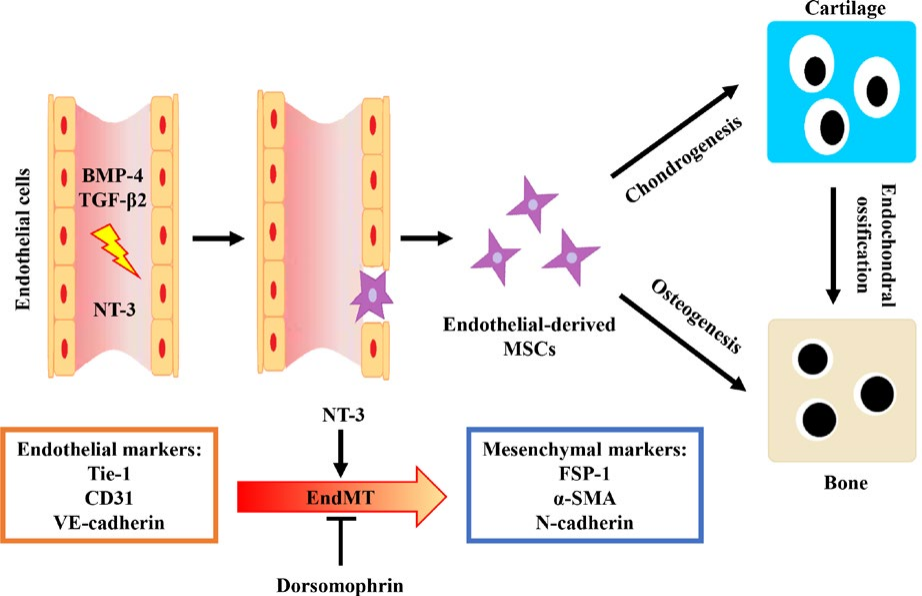
Schematic representation of how Neurotrophin‐3 (NT‐3) promotes heterotopic ossification (HO) by modulating endothelial‐mesenchymal transition (EndMT). Vascular endothelial cells immediately undergo EndMT in response to inflammatory BMP or TGF signals after injury. NT‐3 can promote the EndMT program and induce endothelium‐derived mesenchymal stem‐like cells, ultimately leading to new HO formation

In conclusion, our study is the first step to identify the potential role of NT‐3 in EndMT induction and HO formation. We reveal that NT‐3 may act as neuroendocrine regulator to promote the pathogenesis of HO formation via modulating EndMT both in vivo and in vitro. Suppression of these NT‐3‐induced effects by dorsomorphin and GNF5837 can obviously decrease HO formation. In this study, our findings shed new light on the role and action mechanism of NT‐3 as a neuroendocrine regulator in the pathogenesis of HO associated with EndMT (Figure 9), which offers potential therapeutic targets for the management of HO.

## CONFLICTS OF INTEREST

The authors confirm that there are no conflicts of interest.
