# Supplementary figures and images for: Neurotrophin‐3 acts on the endothelial‐mesenchymal transition of heterotopic ossification in rats
Source: J Cell Mol Med. 2019 Jan 22;23(4):2595–609. doi: 10.1111/jcmm.14150 (PMC6433730; doi:10.1111/jcmm.14150)

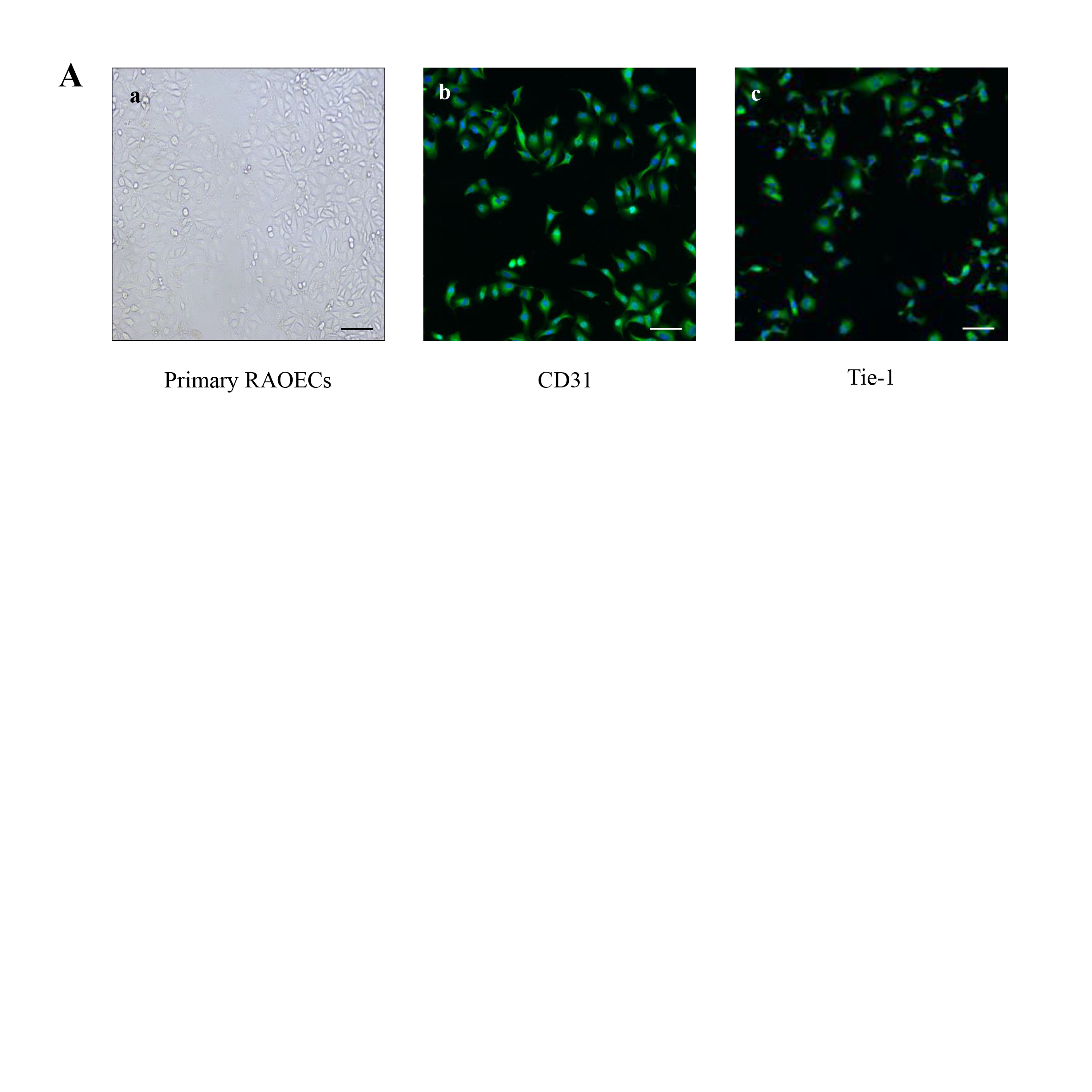

Supplement: Supplementary file 1 [file JCMM-23-2595-s001.tif]
