# Supplementary material for: Neurotrophin‐3 acts on the endothelial‐mesenchymal transition of heterotopic ossification in rats
Source: J Cell Mol Med. 2019 Jan 22;23(4):2595–609. doi: 10.1111/jcmm.14150 (PMC6433730; doi:10.1111/jcmm.14150)
Supplement: Supplementary file 2 [file JCMM-23-2595-s002.docx]

**Table 1.** Primer sequences for qRT-PCR analysis

| **Gene** | **Forward primer (5’-3’)** | **Reverse primer (5’-3’)** |
| --- | --- | --- |
| GAPDH | GGCATTGCTCTCAATGACAA | TGTGAGGGAGATGCTCAGTC |
| NT-3 | TAAAGAAGCCAGGCCAGTCA | AGTCAGTGCTCGGACGTAGG |
| TrkC | GGTTCCAGCTCTCTAACACA | CAGCACCCCAGCATGACATC |
| Tie-1 | AGAGCTGTCAGGAACAGTGC | GGAGTCGAGGTGCAGTCAAA |
| CD31 | CTTCACCATCCAGAAGGAAGA | CACTGGTATTCCATGTCTCTG |
| VE-cadherin | ATCTTCAAGCCGTCCTGTGTG | TGAGGTTTGATCCGCATGATC |
| FSP-1 | TCTTGGTTTGATCCTGACTGCT | CCTGTTGCTGTCCAAGTTGC |
| α-SMA | TGGTGGAAACCCACAACGAA | ACACAGAGATCCGCAGTCCT |
| N-cadherin | CCCAGCGGTGGTTATGACTT | CGGCCACCATCTTGAGACTT |
| Sox9 | AGTACCCGCATCTGCACAAC | ACTTGTAATCGGGGTGGTCT |
| Runx2 | GTCGTCAGACCGAGAAGTGG | TCAAGTTCGAGGAAGCCGTG |
| OCN | CGCGTAAACGCCCTTTTGAT | AGTCTTGCAGCACCCGTAAA |
